# TMEM10 Promotes Oligodendrocyte Differentiation and is Expressed by Oligodendrocytes in Human Remyelinating Multiple Sclerosis Plaques

**DOI:** 10.1038/s41598-019-40342-x

**Published:** 2019-03-05

**Authors:** Omar de Faria, Ajit S. Dhaunchak, Yasmine Kamen, Alejandro D. Roth, Tanja Kuhlmann, David R. Colman, Timothy E. Kennedy

**Affiliations:** 1 Department of Neurology and Neurosurgery, Montreal Neurological Institute, McGill University, 3801 University St., Montreal, Quebec H3A 2B4 Canada; 2 Institute of Neuropathology, University Hospital Münster, D-48149 Münster, Germany; 3 Departamento de Biología, Facultad de Ciencias, Universidad de Chile, Santiago, Chile

## Abstract

Oligodendrocyte precursor cells (OPCs) differentiate during postnatal development into myelin-forming oligodendrocytes, in a process distinguished by substantial changes in morphology and the onset of myelin gene expression. A mammalian-specific CNS myelin gene, *tmem10*, also called *Opalin*, encodes a type 1 transmembrane protein that is highly upregulated during early stages of OPC differentiation*;* however, a function for TMEM10 has not yet been identified. Here, consistent with previous studies, we detect TMEM10 protein in mouse brain beginning at ~P10 and show that protein levels continue to increase as oligodendrocytes differentiate and myelinate axons *in vivo*. We show that constitutive TMEM10 overexpression in the Oli-neu oligodendroglial cell line promotes the expression of the myelin-associated genes MAG, CNP and CGT, whereas TMEM10 knock down in primary OPCs reduces CNP mRNA expression and decreases the percentage of MBP-positive oligodendrocytes that differentiate *in vitro*. Ectopic TMEM10 expression evokes an increase in process extension and branching, and blocking endogenous TMEM10 expression results in oligodendrocytes with abnormal cell morphology. These findings may have implications for human demyelinating disorders, as oligodendrocytes expressing TMEM10 are detected in human remyelinating multiple sclerosis lesions. Together, our findings provide evidence that TMEM10 promotes oligodendrocyte terminal differentiation and may represent a novel target to promote remyelination in demyelinating disorders.

## Introduction

Myelin is a multi-layered lipid-rich membrane that insulates axons. In myelinated fibers, axonal depolarization is confined to the node of Ranvier and, as a result, action potentials rapidly propagate along the axon in a “saltatory” manner^1^. In the central nervous system (CNS), myelin is produced by oligodendrocytes. During differentiation, oligodendrocyte precursor cells (OPCs) elaborate an intricate network of cellular processes, with dynamic leading edges that contact and ultimately ensheath axons^2,3^. Differentiating oligodendrocytes activate a program of myelin gene expression, expressing myelin associated genes such as 2′,3′-Cyclic-Nucleotide 3′-Phosphodiesterase (CNP), UDP-galactose:ceramide galactosyl transferase (CGT), Myelin Associated Glycoprotein (MAG), Proteolipid Protein 1 (PLP) and Myelin Basic Protein (MBP)^3,4^.

Multiple regulatory mechanisms, both external and internally driven, cooperate to control oligodendrocyte differentiation^5^. Part of this regulation is in place to ensure the temporal correlation of oligodendrocyte differentiation and axon maturation. Premature OPC differentiation is prevented by the activation of inhibitory transcription factors such as Sox5, Sox6, Hes5, Id2 and Id4^6–8^. These inhibitory transcription factors are regulated downstream of ligands presented on the axonal surface, such as Lingo-1 and Jagged-1, and by soluble molecules secreted by neurons (e.g. adenosine) and neuronal activity^9–13^. On the oligodendrocyte side, the expression of several transcription factors and other regulatory molecules are necessary for OPC differentiation. Pro-differentiation factors include Olig2, Sox10, Myelin regulatory factor (Myrf) and miRNAs miR-219 and miR-338^14–18^. Although multiple mechanisms have been reported that regulate oligodendrocyte differentiation, others remain to be uncovered and a comprehensive picture of developmental myelination is currently unavailable. Identifying such mechanisms is paramount, as they represent potential therapeutic targets to promote remyelination in human disorders where myelin is lost, such as Multiple Sclerosis (MS).

Transmembrane Protein 10, or TMEM10 (also known as Opalin), is a mammalian-specific type-1 transmembrane glycoprotein expressed by oligodendrocytes but not Schwann cells. The first intron of the TMEM10 gene contains a conserved transcriptional enhancer that directs TMEM10 expression to white matter in the CNS^19^. TMEM10 expression is lost following genetic ablation of oligodendrocytes^20^, and *in situ* hybridization and immunohistochemical analyses have confirmed that, *in vivo*, TMEM10 is expressed in the white matter by oligodendrocytes, with TMEM10 protein enriched in soma, processes and non-compact myelin^20,21^. Immunolabelling of OPCs in culture showed that TMEM10 is detected as soon as 2 DIV, in O4 positive cells, where it appears closely associated with F-actin filaments^22^. In the mouse brain, TMEM10 mRNA is first detected at ~P9, coincident with oligodendrocyte differentiation and initiation of axon myelination in the CNS^20,21^. Strikingly, TMEM10 was the most upregulated transcript detected during differentiation of the OPC-like Oli-neu cell line, an *in vitro* model of early oligodendrocyte differentiation^22^. These observations suggest that TMEM10 may influence OPC differentiation and myelination during development. That TMEM10 is not essential for myelination *in vivo*, as shown by Yoshikawa and colleagues^23^, is expected, as no TMEM10 homologue has been identified in myelinating non-mammalian vertebrates^19^. We hypothesize that TMEM10 may be an evolutionary novelty that contributes in a more subtle way to the elaboration of myelin in mammals.

Here, we generated and characterized a novel TMEM10 specific antibody. Consistent with past studies^20,21^, we detect TMEM10 protein in lysates of P10 mouse brain and show that expression increases as OPCs differentiate *in vivo*. Overexpressing TMEM10 in Oli-neu cells increased membrane extension and induced the expression of myelin-associated genes, while TMEM10 knock down in primary OPCs reduced expression of CNP and MBP, and resulted in oligodendrocytes with abnormal cell morphology. Furthermore, we detect TMEM10 immuno-positive oligodendrocytes in human remyelinating MS plaques. Our findings provide evidence that TMEM10 promotes OPC differentiation and suggest that targeting TMEM10 signaling in oligodendrocytes may promote remyelination in demyelinating disorders.

## Results

### TMEM10 is expressed during oligodendrocyte differentiation and myelination *in vivo*

**Figure 1:**
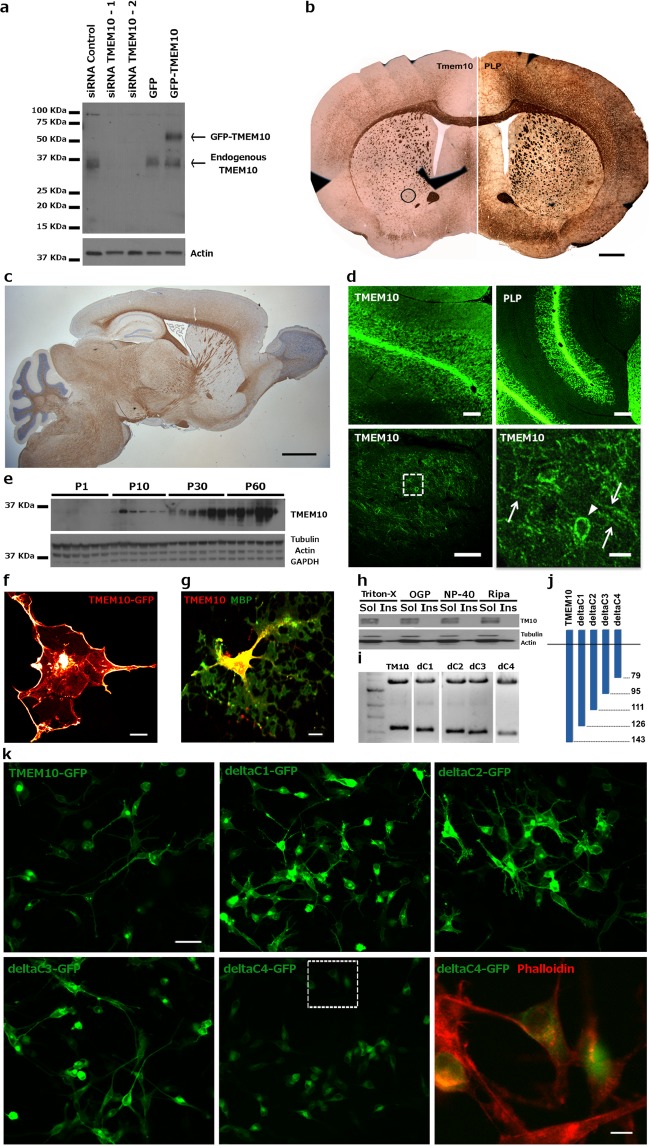
TMEM10 expression profile – (**a**) Validation of TMEM10 antibody. One major band corresponding to endogenous TMEM10 was detected at ~36 KDa. An ~60 KDa band was detected in OPCs transfected with a GFP-TMEM10 expression construct. **(b)** Immunohistochemistry for TMEM10 and PLP on coronal and **(c)** for TMEM10 on sagittal sections of adult mouse brain. Scale bars correspond to 0.8 mm and 1.5 mm, respectively. **(d)** Immunohistochemistry for TMEM10 and PLP on horizontal sections of P35 mouse brain. The area demarcated in the bottom left image is enlarged on the right. Arrowheads indicate the distribution of TMEM10 protein in a cell body, while arrows indicate protein present in cell processes. Scale bars: 350 µm, 300 µm, 90 µm and 20 µm. **(e)** Western blot analysis of a developmental time course of expression in mouse hippocampus. TMEM10 protein was first detected at P10. **(f)** Oli-neu cells were transfected with an expression construct encoding GFP-TMEM10 and F-actin labelled with phalloidin (red). GFP signal was detected on the plasma membrane and accumulated along leading edges of extending processes. **(g)** Primary OPCs were cultured for 3 days and stained for TMEM10 and MBP. TMEM10 protein was distributed along the cell body, primary processes and tips of processes, but not associated with MBP positive membranes. Scale bar: 10 µm. **(h)** Western blot analysis of TMEM10 in soluble and insoluble fractions following extraction of Oli-neu cells with different detergents. Actin was probed as a control for detergent-soluble and insoluble fractions. **(i)** Agarose gel electrophoresis showing C-terminal truncated TMEM10 fragments used to transfect Oli-neu cells. The uncut gel is shown in Supplemental Fig. 2**(j)** Schematic representation of truncated TMEM10 fragments. Numbers on the right indicate the position of the last amino acid encoded by the truncated TMEM10 fragment relative to the full-length protein. **(k)** Oli-neu cells were transfected with GFP-TMEM10 or different C-terminally truncated GFP-TMEM10 fragments. The area demarcated in the bottom middle image is enlarged in the lower right panel. GFP-deltaC4TMEM10 was not detected in cellular processes, but instead accumulates in the cytoplasm. Scale bars: 35 µm and 8 µm.

*In vitro*, TMEM10 is expressed during relatively early stages of OPC differentiation, whereas *in vivo*, TMEM10 is first detected in brain sections of P7-P12 mice, depending on the brain region^20,22^. To further investigate TMEM10 expression and function during development, we generated a new TMEM10 antibody. Western blot analysis using this antibody detected one band at the expected size of ~36 KDa in lysates derived from rat OPC cultures transfected with a control siRNA, but not in lysates of cells transfected with two different TMEM10 siRNAs (Fig. 1a). In addition, lysates of OPCs electroporated with a plasmid encoding GFP-TMEM10 showed an additional TMEM10 band with the expected shift in molecular weight (Fig. 1a), supporting the conclusion that this antibody is specific for TMEM10.

Using our validated TMEM10 antibody, we performed immunohistochemical analysis of brain sections of P35 mouse and observed readily detectable TMEM10 immunoreactivity in the corpus callosum and striatum, in a distribution similar to the major myelin protein PLP (Fig. 1b–d). Strong TMEM10 immunoreactivity was also detected in cerebellar white matter where it was associated with cell bodies and processes (Fig. 1d, arrowhead and arrows, respectively). These observations are in agreement with previous studies that examined TMEM10 expression in the mouse brain^20,21,23,24^. Next, we investigated the temporal pattern of TMEM10 expression. Western blot analysis of lysates from mouse hippocampus (Fig. 1e) and cerebellum (data not shown) detected initial TMEM10 expression at P10, with protein levels increasing up to P60. We conclude that TMEM10 expression is upregulated in the mouse brain during oligodendrocyte differentiation and myelination *in vivo*.

We then examined the subcellular distribution of TMEM10 protein. GFP-TMEM10 expressed in HEK293 cells was detected at the plasma membrane, enriched in the leading edge of extending processes (Fig. 1f, arrowheads). Immunolabelling of endogenous protein in differentiating OPC cultures revealed TMEM10 localized to oligodendrocyte cell bodies, primary processes and process tips, but not MBP positive compact myelin-like membranes (Fig. 1g). Consistent with the absence of TMEM10 from compact myelin, protein extraction experiments using increasingly stringent detergents showed that TMEM10 was completely solubilized by mild, non-ionic detergent extraction (Fig. 1h). This pattern of cellular distribution is consistent with potential contributions of TMEM10 to process extension and branching and suggests that TMEM10 may influence extending oligodendrocyte protrusions. Interestingly, TMEM10 appears to overlap with enrichments of actin filaments in cell processes^22^ and its intracellular domain contains several putative serine/threonine and tyrosine phosphorylation sites^21^.

Mutational studies have previously shown that glycosylation within the N-terminal extracellular domain is necessary for the correct localization of TMEM10 to the plasma membrane^21^. To examine if there is additional regulation of TMEM10 expression at the cell surface we deleted intracellular segments of TMEM10 sequence (Fig. 1i–k). We found that deletion of amino acids 79–143, but not 95–143, results in cytoplasmic accumulation of TMEM10, suggesting that the segment composed of amino acids 79–95 is necessary for the appropriate presentation of TMEM10 at the cell surface (Fig. 1k). Interestingly, this fragment contains three putative phosphorylation sites, implying that TMEM10 trafficking or localization at the cell surface may be regulated by phosphorylation. Our findings, in combination with data from Yoshikawa *et al*. (2008), suggest that presentation of TMEM10 on the plasma membrane is tightly regulated and that mechanisms controlling TMEM10 localization require an intact intracellular domain.

### TMEM10 regulates myelin gene expression

The observation that TMEM10 expression is sharply upregulated during early postnatal development led us to investigate whether TMEM10 is involved in OPC differentiation. TMEM10 is the most upregulated transcript detected during differentiation of the Oli-neu OPC-like cell line^22^. We therefore investigated the possible functional contribution of TMEM10 expression to Oli-neu cell differentiation. Oli-neu cells cultured in 3% horse serum exhibited bipolar morphology and weak O4 staining. In contrast, cells maintained for 48 hrs in serum-free medium supplemented with either 10% neuron-conditioned medium (NCM) or 1 mM cAMP extended long and branched processes (Sup Fig. 1A), resembling immature pre-myelinating oligodendrocytes (Sup Fig. 1A, right panel). Western blot analyses of Oli-neu cell lysates indicated that differentiated Oli-neu cells, but not cells cultured in the presence of serum, expressed the oligodendrocyte marker PLP^3,4^ (Supplementary Fig. S1B).

To investigate the involvement of TMEM10 in Oli-neu differentiation, we generated an Oli-neu cell sub-line that constitutively overexpresses GFP-TMEM10 (Supplementary Fig. S1C). Analysis of Oli-neu growth parameters showed that “TMEM10 Oli-neu” exhibited a significantly reduced growth rate in comparison to wild-type (WT) cells, suggesting that Oli-neu proliferation may be slowed by TMEM10 expression, perhaps by promoting differentiation (Supplementary Fig. S1D; daily growth was 193.4 ± 34.5% for WT oli-neu and 95.4 ± 11.4% for TMEM10 Oli-neu).

**Figure 2:**
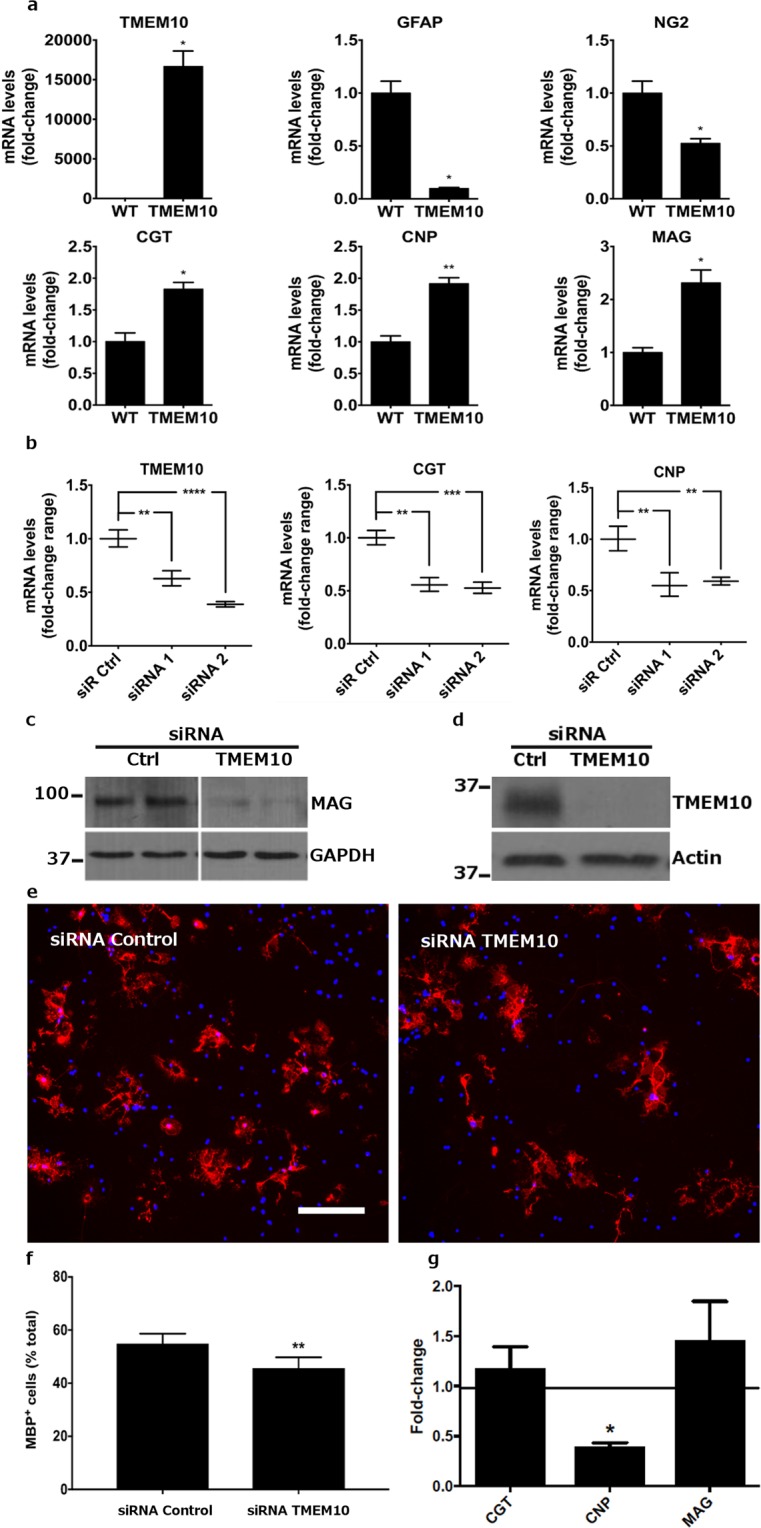
TMEM10 promotes myelin gene expression – (**a)** qPCR analysis of lysates derived from WT and TMEM10 Oli-neu cells. Graphs show mean ± SEM. *p < 0.05; **p < 0.01 (Student’s t-test). **(b)** Oli-neu cells were transfected with control or TMEM10 siRNA and induced to differentiate. qPCR analysis shows reduced levels of CNP and CGT transcripts following TMEM10 knock down. Two experiments, performed in duplicate and analyzed by semi-quantitative RT-PCR, were confirmed in a third independent experiment analyzed by qPCR (shown here). Graphs show mean ± SEM. *p < 0.05 (Student’s t-test). **(c)** Oli-neu cells were transfected as in **(b)** and protein in whole cell lysates examined by western bot analysis for MAG expression. The blot is representative of 2 independent experiments. **(d)** Primary OPCs were electroporated with control and TMEM10 siRNA and cultured for 5 days in differentiating medium. Western blot analysis of protein lysates show TMEM10 knock down. **(e**,**f)** The percentage of MBP positive cells relative to the total number of DAPI-positive nuclei was scored. Scale bar: 35 µm. Twelve independent experiments, performed in triplicate, were analyzed. Graph shows mean ± SEM. ** *p* < 0.01 (paired Student’s t-test). **(g)** mRNA levels for myelin associated genes following TMEM10 knock down in OPCs were analysed by qPCR. Graph shows fold-change expression. *p < 0.05 (paired Student’s t-test, 2–3 independent experiments).

Oli-neu cells activate a program of myelin gene expression when induced to differentiate (Supplementary Fig. S1B). We therefore investigated if TMEM10 overexpression alone could induce a similar change in Oli-neu gene expression. qPCR analysis of myelin gene transcripts showed that early oligodendrocyte markers CNP, MAG and CGT (Fig. 2a), but not late markers MBP and PLP (data not shown) were upregulated in the TMEM10 Oli-neu cells in comparison to WT cells. Non-oligodendroglial genes such as GFAP and OPC markers such as NG2 were markedly downregulated in TMEM10 Oli-neu cells (Fig. 2a). We conclude that TMEM10 overexpression is sufficient to increase the expression of OPC differentiation markers, even in culture conditions that promote proliferation and inhibit differentiation^13^.

To determine if TMEM10 is required for Oli-neu cells to express myelin genes, we conducted knock down experiments in which Oli-neu cells were transfected with control or TMEM10 siRNAs and induced to differentiate in serum-free medium supplemented with 10% NCM. Two different siRNAs targeting TMEM10 successfully downregulated levels of TMEM10 transcript in Oli-neu cells that were cultured in differentiating conditions (Fig. 2b, left panel). To further demonstrate TMEM10 knock down, we transfected TMEM10 Oli-neu cells with TMEM10 siRNAs and conducted western blot analysis on lysates derived from transfected cells. We found that either TMEM10 siRNA reduced GFP-TMEM10 protein levels in comparison to control siRNA (data not shown).

When cultured in serum-free 10% NCM medium, Oli-neu cells differentiated and upregulated the expression of myelin genes (Supplementary Fig. S1). We therefore performed qPCR analysis of myelin gene expression of differentiating Oli-neu cells that were transfected with control and TMEM10 siRNAs. CNP and CGT transcript levels were significantly reduced in differentiating Oli-neu cells transfected with TMEM10 siRNAs in comparison to control levels (Fig. 2b). We also performed western blot analysis of lysates derived from siRNA-transfected cells. Differentiating Oli-neu cells transfected with TMEM10 siRNAs expressed significantly lower levels of MAG protein in comparison to controls (Fig. 2c). These findings support the conclusion that TMEM10 is required for normal expression of myelin genes during NCM-induced Oli-neu differentiation.

As TMEM10 overexpression and knock down experiments in Oli-neu cells suggested that TMEM10 regulates myelin gene expression, we next investigated if TMEM10 knock down could affect the percentage of MBP positive cells in primary cultures of differentiating OPCs. Primary OPCs were isolated from the neonatal rat brain and electroporated with control and TMEM10 siRNA followed by 5 days of culture in Sato medium. Western blot analyses of lysates derived from electroporated cells reveal substantially reduced levels of TMEM10 protein in TMEM10 siRNA-transfected cells compared to controls (Fig. 2d).

Immunostaining these cultures for the mature oligodendrocyte marker MBP and scoring the percentage of MBP-positive cells among the total number of DAPI-positive cells revealed that in TMEM10 siRNA-electroporated OPC cultures, the percentage of MBP-expressing cells was reduced by ~17% compared to control siRNA electroporated cells (Fig. 2e,f and Supplemental Fig. S3; 54.91 ± 5.3% in control siRNA and 45.66 ± 6.1% in TMEM10 siRNA), providing evidence that TMEM10 facilitates OPC differentiation. In these experiments, we also detected a significant reduction in total cell number, although the decrease in the percentage of MBP positive cells and the reduction in cell number were not positively correlated (slope = −0.277, p = 0.193, 12 experiments). In addition, we measured the mRNA levels of selected myelin genes following TMEM10 knock down in OPCs. While CGT and MAG were not changed, CNP levels were decreased by ~50% following disruption of TMEM10 expression (Fig. 2g). Taken together, our experiments suggest that TMEM10 regulates myelin gene expression during OPC differentiation *in vitro*.

### TMEM10 regulates membrane extension and cell morphology

**Figure 3:**
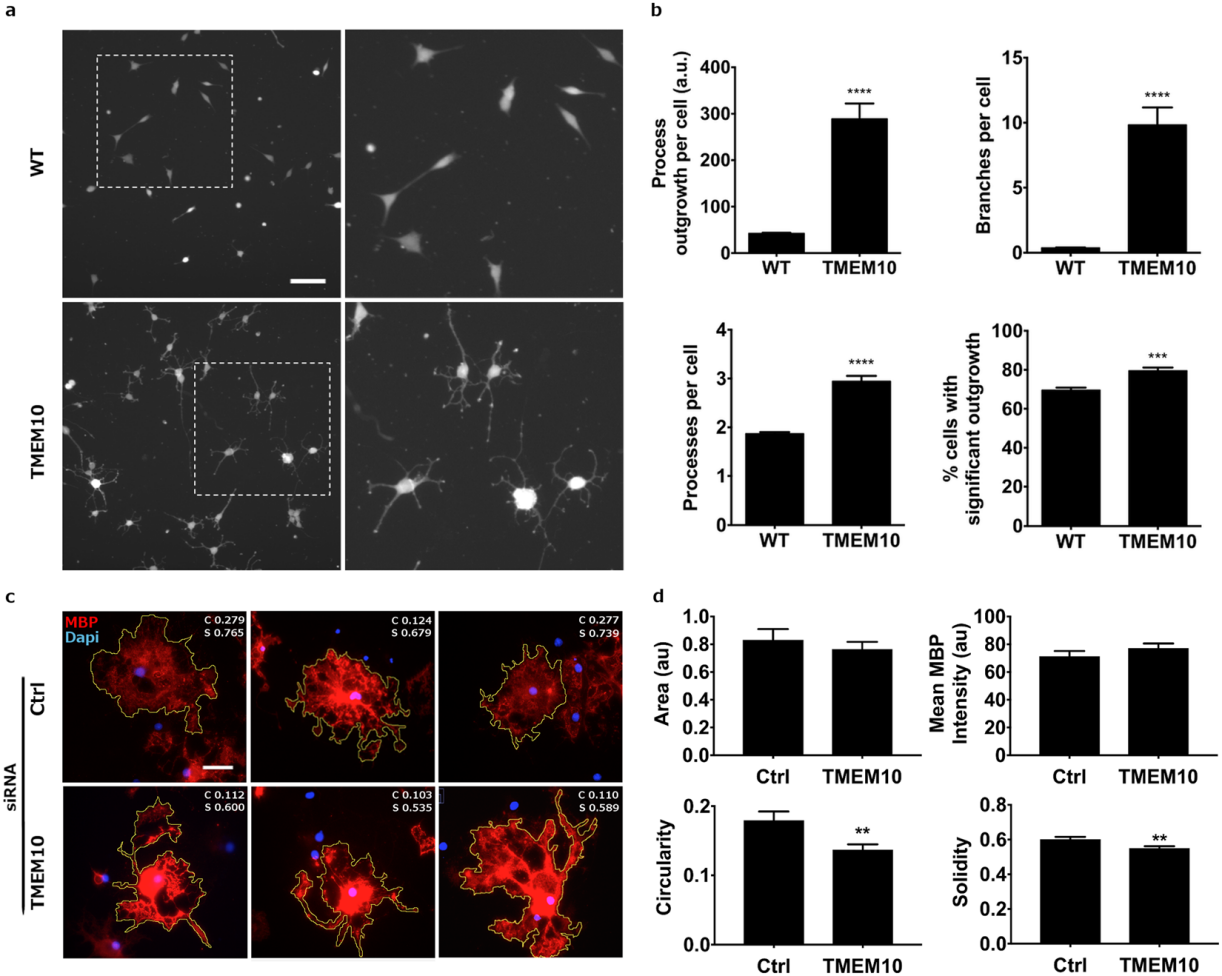
TMEM10 influences membrane extension and oligodendrocyte cell morphology – (**a)** WT and TMEM10 Oli-neu cells were cultured for 2 days and stained with Cell Tracker, a membrane labeling fluorescent dye. Regions demarcated on the left are enlarged in the right hand panels. Scale bar: 60 µm. **(b)** Process length, number of branches, number of processes and percentage of cells with significant outgrowth were measured. Graphs show mean ± SEM. Ninety fields from two independent experiments performed in triplicates were analyzed. ***p < 0.001; ****p < 0.0001 (Student’s t-test). **(c)** Primary OPCs were transfected with control and TMEM10 siRNAs, cultured for 5 days in differentiating medium, fixed and stained for MBP. 100–150 cells from six independent experiments were randomly selected and individually outlined using the free selection tool of ImageJ software. Representative images are shown. Measurements of cell circularity (c) and solidity (s) are shown on the top right corner of each image. Scale bar: 3.5 µm. **(d)** Quantification of total membrane area, mean MBP fluorescence, cell circularity and solidity. Graphs show mean ± SEM. **p < 0.01 (Student’s t-test).

During oligodendrocyte differentiation, changes in gene expression are accompanied by membrane extension and substantial remodeling of cell morphology. As Oli-neu cells extend long and branched processes during induced differentiation, we determined if TMEM10 overexpression alone could induce a similar change in Oli-neu morphology. WT Oli-neu cells cultured in the presence of serum extended few and short processes, as expected. In contrast, the gain-of-function TMEM10-Oli-neu cells cultured in the same conditions extended many processes that were longer and more branched (Fig. 3a). On average, the processes of TMEM10 expressing Oli-neu cells were nine times longer and more than thirty times more branched compared to WT Oli-neu. Further, the substrate adherence of TMEM10-Oli-neu cells was not significantly different from WT, suggesting that this difference was not due to grossly altered cell adhesion or viability (59% WT and 74% of TMEM10-Oli-neu cells adhered to uncoated cell culture plastic plates, p = 0.18; and 97% WT and 96% TMEM10-Oli-neu cells adhered to PLL coated cell culture plates, p = 0.41). These findings indicate that TMEM10 expression triggers substantial changes in morphology, even in the presence of serum (Fig. 3b).

As overexpression experiments in Oli-neu cells provided evidence that TMEM10 may regulate membrane extension during differentiation, we next investigated if knocking down TMEM10 expression in primary cells would affect oligodendrocyte cell morphology. When cultured in Sato medium for 5 days, OPCs differentiate and extend myelin-like membranes that are enriched in MBP. Therefore, we immunostained for MBP in OPC cultures transfected with control and TMEM10 siRNAs and performed shape descriptor analysis to quantitatively describe the morphology of differentiating OPCs (see Methods section). Total membrane area and MBP fluorescence were not affected by TMEM10 knock down, indicating that, in primary cells, TMEM10 is not required for the extension of MBP positive membranes. In contrast, measurements of cell circularity and solidity were significantly reduced compared to controls, indicating abnormal membrane extension in the absence of TMEM10 (Fig. 3c,d). These findings indicate that TMEM10 regulates membrane extension and oligodendrocyte cell morphology and support the conclusion that TMEM10 contributes to terminal differentiation of OPCs *in vitro*.

### TMEM10 expression in human MS plaques

**Figure 4:**
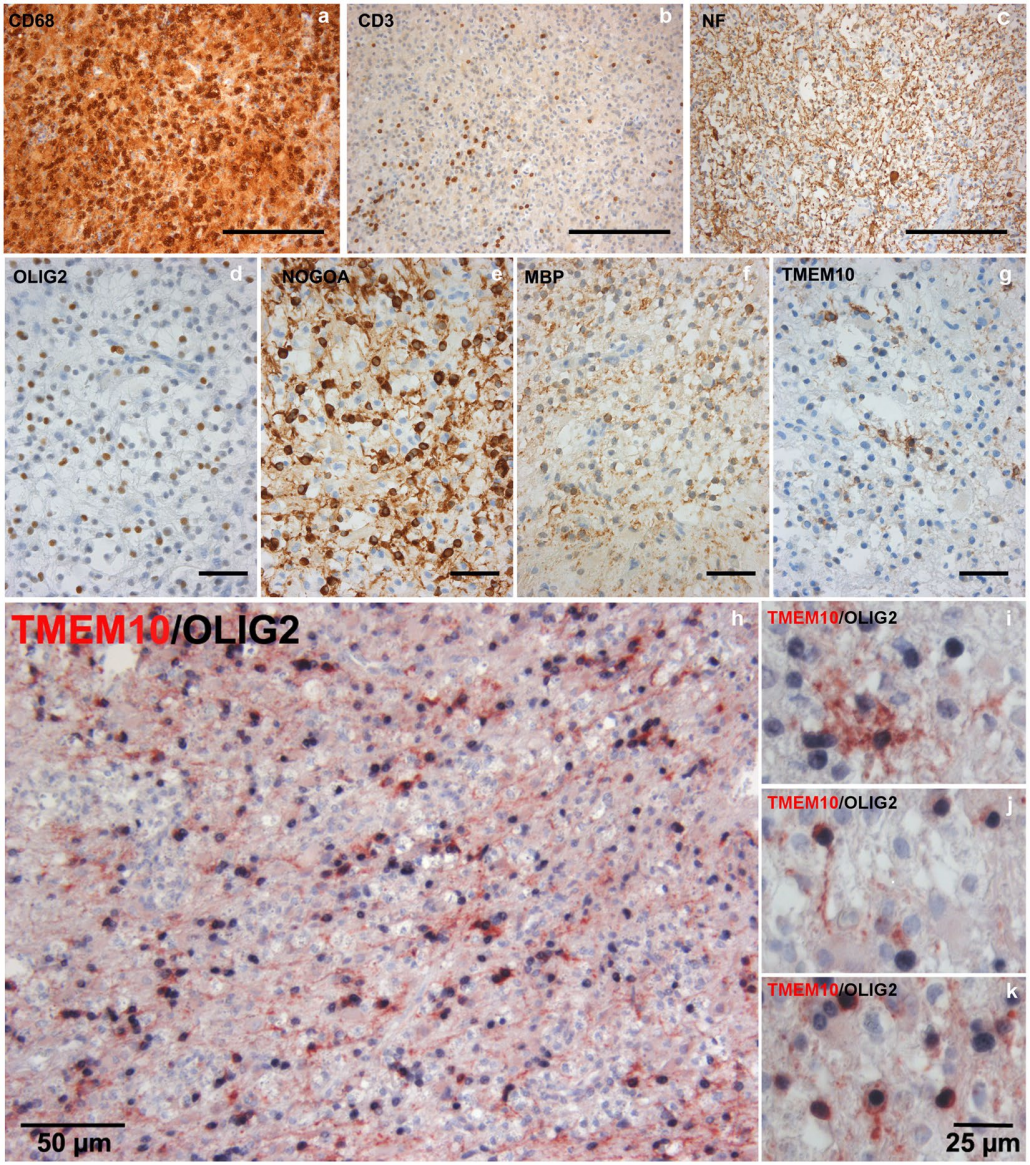
TMEM10 is expressed by oligodendrocytes in remyelinating MS plaques –(**a**–**f)** Brain tissue samples containing an inflammatory demyelinating lesion consistent with MS were stained for CD68 and CD3 (markers of infiltrating immune cells), Neurofilament (axons), Olig2 (oligodendrocyte lineage cells), and TMEM10. NOGOA and MBP expression indicate ongoing remyelination in these plaques. **(g)** A subset of cells with oligodendroglial morphology expresses TMEM10. **(h–k)** Double staining for TMEM10 and Olig2 confirms that TMEM10 expressing cells are oligodendrocytes. Scale bars correspond to 200 µm in (**a**,**b**), 100 µm in (**c**), 50 µm in (**d**) through (**h**) and 25 µm.

MS is an autoimmune disorder in which CNS myelin is attacked by the immune system. Although OPCs in the adult CNS are initially able to differentiate and remyelinate demyelinated regions, remyelination efficiency decreases as MS progresses, likely due to impairment of OPC differentiation. Currently, approved therapies that improve remyelination efficiency are not available and the elucidation of new mechanisms that regulate OPC differentiation and myelin formation may contribute to the development of remyelinating therapies. Our *in vitro* data suggested that TMEM10 promotes OPC differentiation. We therefore investigated the possibility that TMEM10 may be expressed during remyelination in MS lesions. To address this, tissue samples from 6 patients with histological findings consistent with the diagnosis of MS were immunolabelled for markers of immune cell infiltration, OPC recruitment, myelin and TMEM10. All sample lesion areas were infiltrated by numerous CD68+ macrophages/microglia and some CD3+ T cells. Axons in the lesion were relatively well preserved, although a few axonal spheroids are visible. Remyelination, as indicated by thin irregularly formed myelin sheaths, was detected in four out of six lesion samples. In five out of six lesions (four lesions with, 1 lesion without, signs of remyelination) we detected TMEM10 positive oligodendrocytes, identified morphologically and by co-immunostaining with an antibody against Olig2 (Fig. 4h–k). In three out of the five lesions (all lesions with signs of remyelination) TMEM10 positive cells were abundant in the sampled lesion area (Fig. 4) whereas in the two other tissue samples few TMEM10 positive cells were observed. However, even in the lesions with relatively high numbers of TMEM10-positive oligodendrocytes only a subset of oligodendrocytes expressed TMEM10, indicated by comparison with other oligodendroglial markers (NogoA, Olig2). In summary, these findings provide evidence that TMEM10 is expressed by oligodendrocytes during remyelination in MS (Fig. 4). Taken together, our results raise the possibility that TMEM10 may be a relevant therapeutic target to overcome remyelination failure in MS.

## Discussion

Despite substantial advances made in the study of oligodendrocyte differentiation, many aspects of this complex process have yet to be elucidated. Here, we provide evidence that the myelin glycoprotein TMEM10 regulates OPC differentiation. During postnatal brain development, we first detected TMEM10 protein at ~P10, with levels continuing to increase during maturation. Increasing TMEM10 expression in undifferentiated Oli-neu cells increased morphological differentiation and transcript levels of myelin associated genes. Conversely, knocking down TMEM10 in differentiating Oli-neu cells decreased myelin gene expression and, in the absence of TMEM10, primary OPCs differentiated into oligodendrocytes with abnormal morphology and reduced MBP expression. These findings support the conclusion that TMEM10 promotes OPC terminal differentiation.

It was recently shown that apparently normal myelin develops in the absence of TMEM10, indicating that TMEM10 is not essential and perhaps functionally redundant for myelin formation *in vivo*^23^. TMEM10 knockouts display normal expression profiles for major myelin proteins and typical myelin domain organization, with no gross abnormalities in myelin ultrastructure, indicating that developmental myelination does not require TMEM10 *in vivo*^23^; however, this study does not rule out possible more subtle influences of TMEM10 on OPC maturation and myelination. Our *in vitro* approach has been informative, revealing a contribution of TMEM10 to OPC differentiation, promoting myelin gene expression and membrane extension. Interestingly, while TMEM10 is specific to mammals, with no obvious non-mammalian evolutionary orthologue^19^, well-developed compact myelin is characteristic of many non-mammalian vertebrate species. This is consistent with a non-essential role for TMEM10 in myelination, but perhaps a more subtle contribution to myelin formation in mammals.

TMEM10 was the most up regulated transcript detected during NCM-induced differentiation of Oli-neu cells^22^. Oli-neu cell differentiation is a model for initial OPC differentiation, suggesting that the onset of TMEM10 expression might similarly occur at early stages of primary OPC development *in vitro*. In agreement with this, TMEM10 expression was detected in primary OPC cultures as early as 2 DIV by cells double positive for the oligodendrocyte markers NG2 and O4. In these cultures, expression persisted thereafter and TMEM10 immunoreactivity strongly labeled differentiating and mature oligodendrocytes that expressed the mature oligodendroctye marker MBP^22^. This temporal pattern of expression was recapitulated in the mouse brain, where we and others have shown that TMEM10 is first detected at approximately one week post-natally and continues to be expressed at high levels at older ages^20,21^ (Fig. 1d). This suggests that in addition to a possible contribution to OPC differentiation, TMEM10 expression may influence the maintenance of the mature oligodendrocyte phenotype, as has been demonstrated for other pro-differentiation factors. For example, conditional deletion of Myrf in adult oligodendrocytes resulted in demyelination and loss of myelin gene expression, indicating that this transcription factor is necessary for both OPC differentiation and maintenance of oligodendrocyte identity in the adult brain^25^. Similar results were obtained in the PNS following late-onset ablation of Krox-20 and Sox10, providing further examples of the persistent expression of differentiation factors contributing to myelin maintenance in the mature nervous system^26,27^.

An aspect of TMEM10 function that has emerged from our findings and is consistent with previous studies^22^ is a putative role regulating cytoskeletal remodeling and morphology. TMEM10 accumulates at the leading edge of membrane extensions and at the tips of processes (Fig. 1f–g), typically overlapping enrichments of F-actin^22^. Following latrunculin A-induced destabilization of F-actin, a fraction of TMEM10 was found to accumulate in the cytoplasm, indicating that localization at the plasma membrane requires F-actin^22^. This phenotype is similar to the distribution we observed when a truncated form of TMEM10 that lacks amino acids 79–143 was expressed in Oli-neu cells, suggesting that appropriate trafficking via a mechanism that engages this intracellular region may require an interaction with F-actin (deltaC4-TMEM10, Fig. 1k). TMEM10 overexpression also induced a striking increase in Oli-neu process length and branching reminiscent of morphological changes associated with Oli-neu differentiation (Fig. 3a,b). Consistent with this phenotype, differentiating primary OPCs in which TMEM10 had been knocked down extended abnormally shaped MBP-positive membranes (Fig. 3c,d). Together, these results provide evidence that TMEM10 influences OPC morphology and suggest that this regulation may involve interactions with the F-actin cytoskeleton.

During postnatal CNS development, the onset of oligodendrocyte differentiation and myelination must be temporally coordinated with axonal maturation. Signals mediating this neuron-glia interaction include protein ligands presented by the axonal plasma membrane^9,10^, as well as soluble molecules secreted by neurons^12,13^. We report that TMEM10 knock down decreased myelin gene expression induced by a combination of NCM treatment and serum removal (Fig. 2b). In addition, TMEM10 overexpression was sufficient to mimic NCM-induced differentiation in serum-free conditions (Figs 2a and 3a,b). These findings imply that TMEM10 is involved in regulatory mechanisms initiated by serum withdrawal or, alternatively, that TMEM10 functions downstream of a neuron-secreted signal to facilitate Oli-neu cell differentiation. Notably, TMEM10 expression is rapidly and strongly upregulated by NCM treatment^22^; however, further studies are required to determine the significance of this mechanism *in vivo*.

The signaling underlying NCM-induced TMEM10 expression is not known; however, it may involve the second messenger cAMP and its effector molecule CREB1. The oligodendrocyte enhancer located within the first intron of the TMEM10 gene contains a CREB1 binding site and increasing the intracellular concentration of cAMP induces TMEM10 expression^19^. Both cAMP and TMEM10 contribute to Oli-neu/OPC differentiation *in vitro*, suggesting that TMEM10 may function downstream of cAMP in a common signaling cascade^28,29^. Further, inhibiting phosphodiesterase, a cAMP degrading enzyme, increases cAMP levels *in vitro* and enhances remyelination *in vivo*, suggesting that TMEM10 might contribute downstream of cAMP during remyelination^30^. Regulatory mechanisms downstream of TMEM10 might depend on the phosphorylation of its intracellular domain, as several putative sites for phosphorylation are present in this region. These include motifs for phosphorylation by MAPK and Calmodulin-dependent protein kinase II^21^. Interestingly, an unbiased proteomic search for calmodulin partners revealed that TMEM10 binds calmodulin^31^. Although an intriguing possibility, it remains to be demonstrated that TMEM10 is phosphorylated by any kinase.

Elucidating mechanisms that regulate oligodendrocyte differentiation, myelin formation, and myelin maintenance is critical as myelin integrity is compromised during aging and in many neurological diseases. Although OPCs in the adult CNS have the capacity to differentiate and regenerate myelin following demyelination, remyelination is limited or fails during aging and in chronic stages of demyelinating disorders, such as in progressive MS^32–34^. A major reason for the failure to remyelinate in these situations is likely impaired OPC differentiation, rather than OPC recruitment or proliferation, as OPCs are often present in chronic MS lesions^35^ and transgenic PDGF-AA expression in aged mice, which promotes OPC proliferation, does not affect the rate of remyelination^36^. Our findings provide evidence that TMEM10 promotes OPC differentiation and suggest that TMEM10 expressed in demyelinated MS lesions may contribute to remyelination. Further investigation will be necessary to define the functional contribution of TMEM10 to remyelination and address the potential of TMEM10 as a novel therapeutic target in MS.

## Methods

### Animals

Sprague Dawley rats were obtained from Charles River laboratories (Quebec, CA). All procedures were performed in accordance with the Canadian Council on Animal Care guidelines for the use of animals in research and approved by the Montreal Neurological Institute Animal Care Committee and the McGill Animal Compliance Office.

### Antibodies

The following antibodies were used in this study: mouse monoclonal anti-α-actin (Sigma); mouse monoclonal antiβ-tubulin III (Cell Signaling); rabbit polyclonal GAPDH (Santa Cruz Biotechnology). Mouse polyclonal anti-PLP was provided by Dr. Marjorie Lees, University of Massachusetts Medical School, Massachusetts, USA. Rabbit polyclonal anti-MAG^37^ and rabbit polyclonal anti-MBP^38^ were made in lab and have been previously published. TMEM10 polyclonal antibody was generated by immunizing rabbits with a peptide antigen corresponding to amino acids 65–142 of rat TMEM10. The specificity of the antiserum was validated by western blot analysis of OPC cultures transfected with control and TMEM10 siRNAs.

### Cell culture

The Oli-neu cell line (provided by Dr. Jacqueline Trotter, University of Mainz, Mainz, Germany) was maintained in DMEM supplemented with N1 (Sigma-Aldrich,) and 3% horse serum (proliferation medium) on PLL-coated tissue culture dishes. Oli-neu differentiation was induced by culturing these cells in serum-free DMEM-N1 medium supplemented with 10% neuron-conditioned medium or 1–2 mM 8Br-cAMP (Sigma-Aldrich) for 48 hrs. Neuron-conditioned medium was collected from E15–17 cortical neurons cultures that were maintained *in vitro* for two weeks. TMEM10-expressing Oli-neu cells were generated by transfecting Oli-neu cells with a GFP-TMEM10 construct. Sequence encoding human TMEM10 was cloned into the pEGFP-N1 plasmid, in which expression is regulated by the CMV promoter. Hygromicin B was used to select cells that stably expressed the construct. After clone isolation, TMEM10 Oli-neu cells were maintained in proliferation medium. To assess cell adhesion and viability, WT and TMEM10 Oli-neu cells were plated on PLL-coated or uncoated cell culture plastic dishes, maintained in culture for 2 hrs, then fixed with 4% PFA, stained with DAPI to mark nuclei, and adherent cells counted.

Rat primary OPC cultures were prepared as previously described^39^. Briefly, mixed glial cultures were generated from P2 rat cortices and maintained for 17 days prior to OPC isolation in DMEM, 10% fetal bovine serum (FBS) and antibiotics. OPCs were isolated using the shake-off method^40^ and plated in Sato medium (DMEM, 5 µg/ml insulin, 100 µg/ml transferrin, 30 nM sodium selenite, 30 nM triiodothyronine, 100 µg/ml penicillin-streptomycin, 2 mM glutamax). For electroporation experiments, OPCs were allowed to recover for 24 hrs after isolation and 12 hrs after electroporation in DMEM, 10% FBS, before medium was changed to Sato medium.

### Growth rate assay

Wild type (WT) and TMEM10 Oli-neu cells were plated at low cell density (1300 cells/cm^2^) and cultured under normal conditions for 2, 24, 48 and 72 hrs. At each time point, cells were fixed with 4% paraformaldehyde (PFA) and stained with the nuclear marker DAPI (4’,6-diamidino-2-phenylindole). Images were taken with an ImageXpress microscope (Molecular Devices) and the number of DAPI-positive nuclei counted using MetaXpress software.

### Immunohistochemistry

Mice were deeply anesthetized with 4X avertin and perfused transcardially with phosphate buffered saline, pH 7.4 (PBS) followed by 4% PFA, PBS. Brains were dissected and post-fixed with 4% PFA, overnight, at 4 °C with gentle shaking and subsequently equilibrated in 30% sucrose for 1 week at 4 °C. After embedding in optimal cutting temperature (OCT) compound (Sakura Finetek), tissue was cut in 20 μm-thick sections using a Leica cryostat. Slides were allowed to dry for 15 min before being stored at −80 °C. For immunohistochemistry, slides were allowed to equilibrate to room temperature (RT) then were washed with PBS 3 times for 5 min per wash. Sections were blocked with 5% BSA, 0.3% Triton-X, PBS for 1 hr, at RT, and incubated overnight, at 4 °C, with primary antibodies diluted in 3% BSA, 0.3% Triton-X, PBS. Sections were then washed with PBS 3 times for 10 min per wash, and incubated overnight, at 4 °C, with the appropriate secondary antibodies diluted in 3% BSA, PBS. PBS wash steps were repeated followed by a brief wash with water. Sections were immediately mounted in mounting media. Images were taken using an Olympus Fluoview confocal microscope.

### Morphological analysis

WT and TMEM10 Oli-sneu cells were cultured under proliferation conditions for 48 hrs. Cells were then fixed with 4% PFA and stained with Cell Tracker dye (ThermoFisher). Images were taken with an ImageXpress microscope (Molecular Devices) and analyzed using MetaXpress software. Measurements taken included process outgrowth; number of branches per cell; number of processes per cell; and percentage of cells exhibiting process outgrowth. For morphological analysis of primary OPCs, cell cultures were fixed in 4% PFA and immunolabeled for MBP. Images were taken using an Axiovert 100 microscope (Carl Zeiss) with a MagnaFire CCD camera (Optronics). 50–100 cells were randomly selected and individually outlined using the selection tool in ImageJ^41^. Mean fluorescence intensity and cell area were measured and multiple shape descriptors calculated using ImageJ. Circularity is defined as the degree to which a particle is similar to a circle, taking into consideration the smoothness of the perimeter and solidity as an inverse measure of the overall concavity of a particle^42^.

### qPCR

Reverse transcription was performed as previously described^39^. For qPCR, 20 ng of cDNA were added to 5 µM primers and 2X SYBR Green PCR master mix (Invitrogen). An Applied Biosystems 7000 thermocycler was used to perform all qPCR reactions. Data analysis was done using the 2^−[delta][delta]Ct^ method^43,44^ and final expression values were normalized to 18S RNA.

### Transfections

Plasmid and siRNA transfections on cell lines were performed using Lipofectamine 2000 and Lipofectamine RNAiMAX (Invitrogen) transfection reagents. Primary OPC electroporation was performed in an Amaxa nucleofactor using the Basic Nucleofactor kit for Primary Mammalian Glial Cells (Lonza). SiRNA sequences targeting TMEM10 were GAGGUGCUCAAGAAGGUCACAUAUA (Thermo Fischer Scientific, RSS360216) for rat, and GAACCAUCGAUGAUGAGCCCGUAGA (Thermo Fischer Scientific, MSS279035) and CACUGAACUUUACACUGCCAUCGAA (Thermo Fischer Scientific, MSS279037) for mouse. MISSION siRNA Universal Negative Control #1 (Sigma) was used as control.

### TMEM10 truncations

Pairs of PCR primers were designed to amplify C-terminally truncated fragments of mouse TMEM10. PCR products were cloned into pEGFP-N1 using BglII/AgeI sites and transformed into *Escherichia coli*.

### Western Blot

Protein lysates were prepared from freshly dissected rodent hippocampus and cerebellum. Isolated tissues were homogenized on ice with Ripa buffer containing protease and phosphatase inhibitors. Equal amounts of protein were resolved by SDS-PAGE and transferred to PVDF membrane (BioRad). Membranes were blocked with 5% milk or 5% BSA, according to the primary antibody manufactures instructions, in TBS containing 0.1% Tween (TBST) for 1 hr at RT and incubated with primary antibodies diluted in 1% milk, TBST, overnight at 4 °C. Membranes were washed three times with TBST and incubated with horseradish peroxidase-conjugated secondary antibodies for 1 hr at RT. Membranes were washed and developed with an Enhanced Chemoluminescence Detection kit (Pierce).

### Human tissue samples and immunohistochemistry

We retrospectively investigated 6 brain biopsies from 6 patients which fulfilled the generally accepted criteria for the diagnosis of multiple sclerosis^45^. All sampled lesion areas were classified as active and demyelinating; in four out of six lesions remyelination was detected^46^. None of the study authors was involved in decision-making with respect to biopsy. The study was approved by the Ethics Committee of the University of Münster, with all methods performed in accordance with the relevant guidelines and regulations, and informed consent for surgery obtained from all patients. Tissue specimens were fixed in 4% PFA and embedded in paraffin. Tissue samples were cut in 4 µm thick sections that were stained with haematoxylin and eosin and Luxol-fast blue. Immunohistochemical staining was performed with an avidin-biotin technique using an automated staining device (DakoLink 48). The primary antibodies were rabbit anti-myelin basic protein (1:1000) (Boehringer Mannheim, Mannheim, Germany), mouse anti-KiM1P (1:5000) (H.-J. Radzun, Department of Pathology, University of Göttingen, Germany), rabbit anti-Olig2 (1:300) (IBL, Spring Lake Park, Minnesota), rabbit anti-Nogo-A (1:750) (Chemicon International, Temecula, CA) and mouse anti-Nogo-A (1:15.000) (11c7, gift from M.E. Schwab, Brain Research Institute, University of Zürich and Department of Biology, Swiss Federal Institute of Technology Zürich, Switzerland), rabbit anti-CD3 (1:100) (Dako), mouse anti-neurofilament (1:1000) (Dako) and rabbit anti-TMEM10 (1: 250) (Abcam).

### Statistical Analysis

Data was analyzed using the GraphPad Prism software. Results were considered significant when *p*-values were *p < 0.05, **p < 0.01 and ***p < 0.001. Statistical tests used are indicated in the figure legends.

## Supplementary information

Supplementary Information

## Data Availability

All data generated or analyzed during this study are included in this published article (and its Supplementary Information files) or are available from the corresponding author on reasonable request.
