# Supplementary material for: TMEM10 Promotes Oligodendrocyte Differentiation and is Expressed by Oligodendrocytes in Human Remyelinating Multiple Sclerosis Plaques
Source: Sci Rep. 2019 Mar 5;9:3606. doi: 10.1038/s41598-019-40342-x (PMC6400977; doi:10.1038/s41598-019-40342-x)
Supplement: Supplementary file 1 — Supplementary Information [file 41598_2019_40342_MOESM1_ESM.pdf]

TMEM10 Promotes Oligodendrocyte Differentiation and is Expressed by Oligodendrocytes in Human Remyelinating Multiple Sclerosis Plaques

Omar de Faria Jr[1], Ajit S. Dhaunchak[1], Yasmine Kamen[1], Alejandro D Roth[1,3],
Tanja Kuhlmann[2], David R Colman[1] and Timothy E. Kennedy[1*]

[1]Department of Neurology and Neurosurgery, Montreal Neurological Institute, McGill University, 3801 University St., Montreal, Quebec, Canada H3A 2B4.

[2]Institute of Neuropathology, University Hospital Münster, D-48149, Münster, Germany.

[3]Departamento de Biología, Facultad de Ciencias, Universidad de Chile, Santiago, Chile

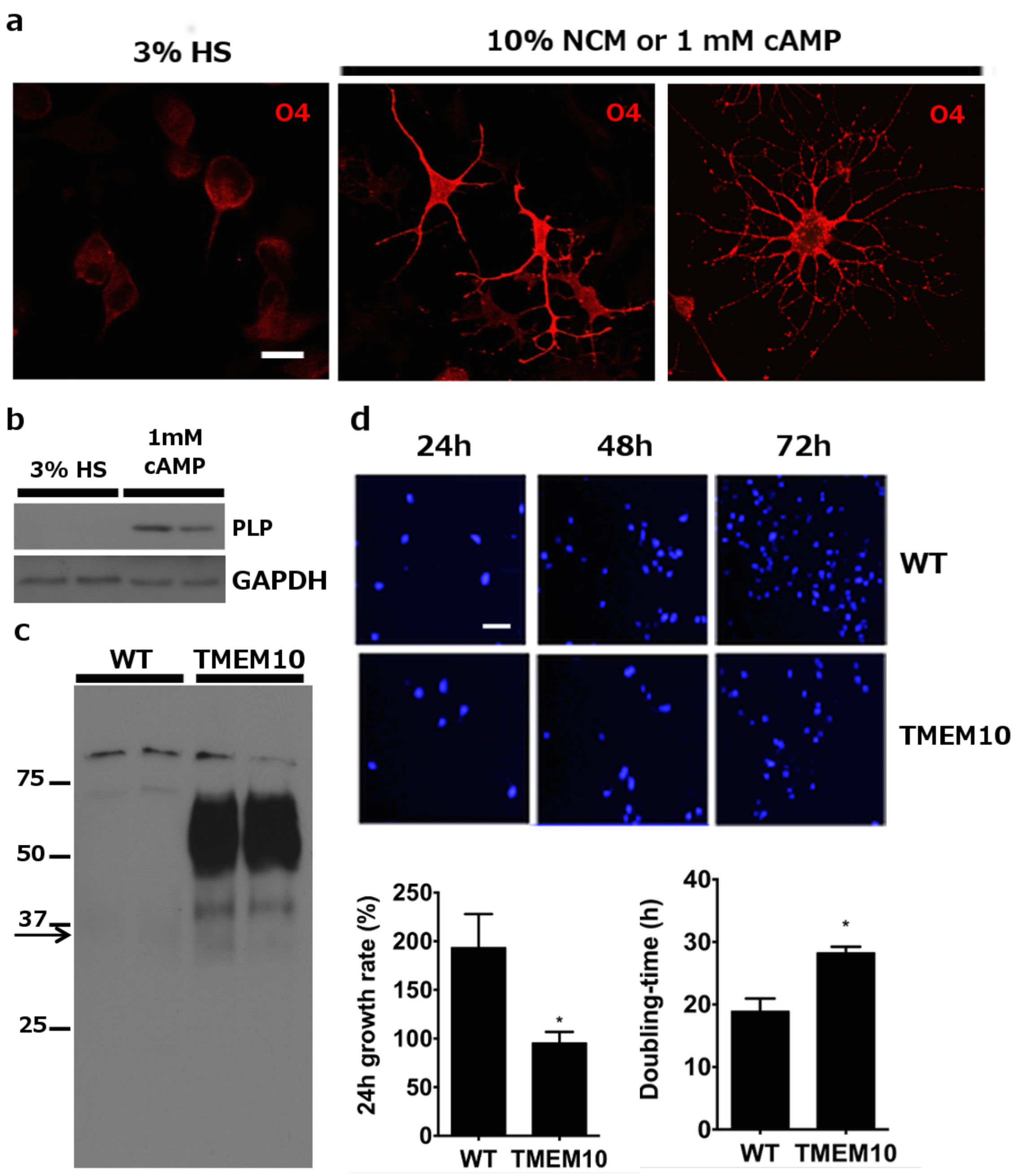

Supplemental Figure 1: The OPC-like cell line oli-neu as a model to study OPC differentiation - (a) O4 staining of oli-neu cells cultured in the presence of 3% horse serum or 10% NCM/1mM cAMP in serum-free medium. **(b)** Western blot analysis cell lysates from oli-neu cells cultured in medium with 3% horse serum or in 1mM cAMP in

serum-free medium. **(c)** Western blot analysis of lysates derived from WT and GFP-TMEM10 expressing oli-neu cells immunoblotted for TMEM10. The arrow marks the expected molecular weight of endogenous TMEM10, ~36 KDa. **(d)** WT and TMEM10 oli-neu cells were monitored for growth rate over 3 days. Cells were plated at the same density on day 0. Graphs show mean ±SEM of three independent experiments. * $p < 0.05$ (student t-test).

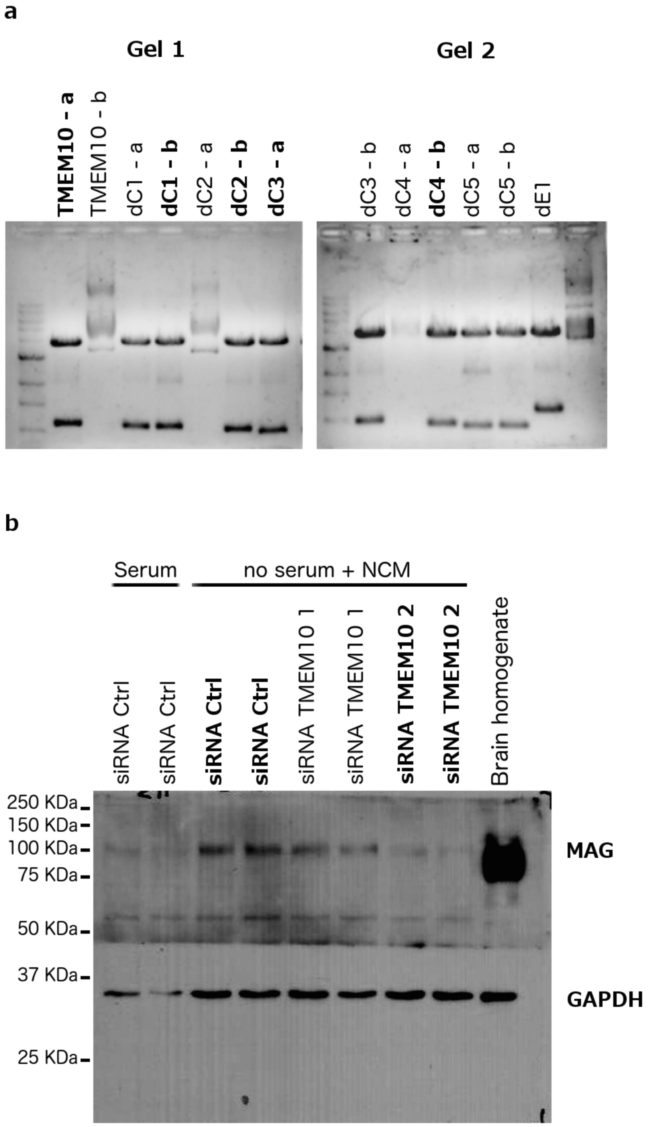

Supplemental Figure 2: Full-length blots – (a) Digested plasmids encoding cloned TMEM10 truncations. The labels in bold (2, 5 and 7 in gel1; and 4 in gel 2) appear in Figure 1i. **(b)** Oli-neu cells were transfected with control or two different TMEM10 siRNAs and cultured in medium with serum or in the absence of serum plus neuronal conditioned medium (NCM). Western blot analyses shows MAG and GAPDH expression. The membrane was split for antibody incubation and the two

halves were placed back together when exposed to film. The lanes in bold (3, 4, 7, 8) appear in Figure 2c.

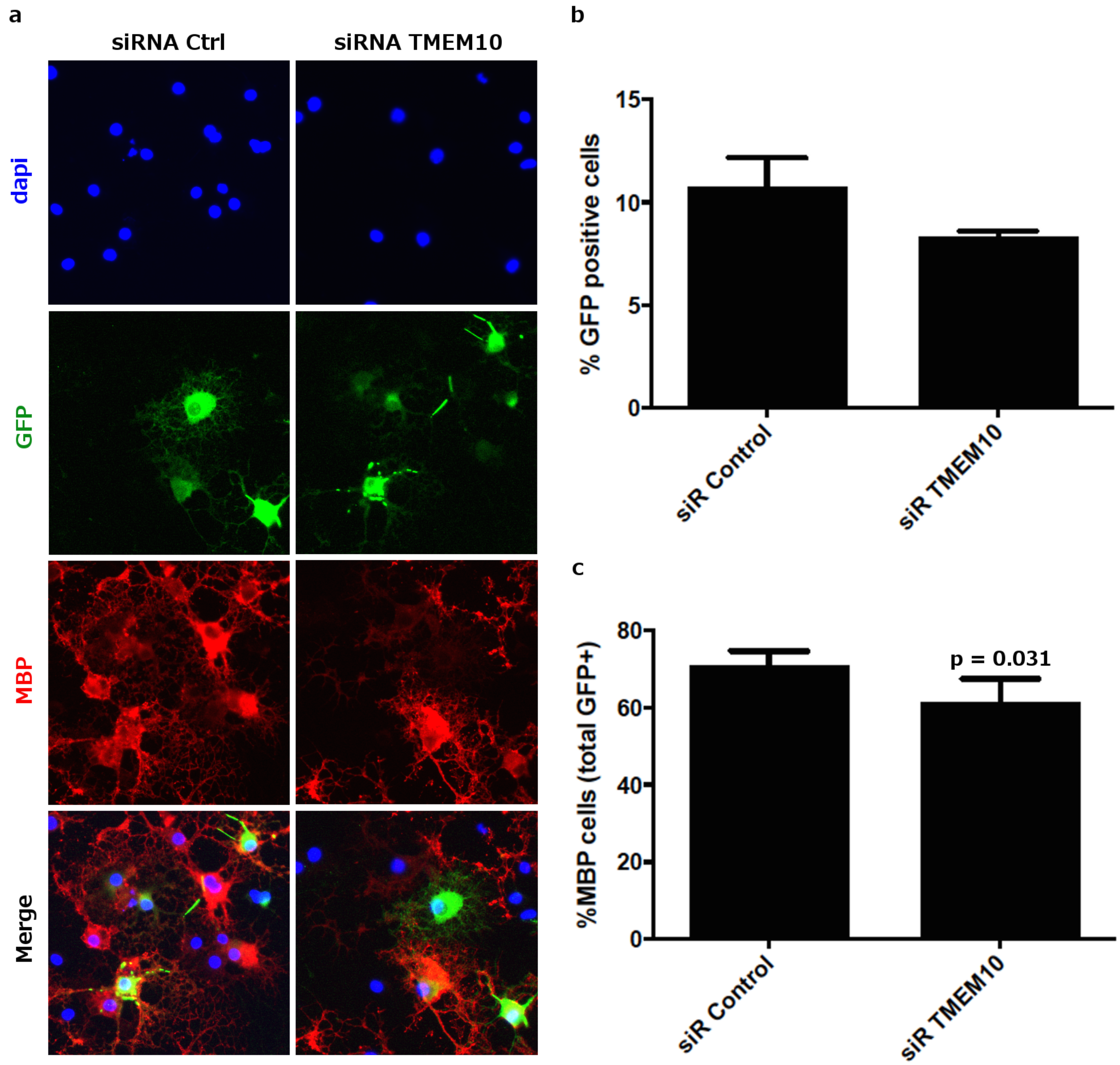

Supplemental Figure 3: TMEM10 knock down decreases the number of MBP-positive OPCs – OPCs were electroporated with a GFP plasmid and either control or TMEM10 siRNA and cultured in differentiation medium for 5 days. **(a,c)** Cultures were immunostained for MBP and the percentage of MBP/GFP-double positive cells scored. P = 0.031, paired Student's one tailed t-test, 3 experiments. **(b)** Approximately 10% of OPCs were transfected by the GFP plasmid.
